# BTLA blockade enhances Cancer therapy by inhibiting IL-6/IL-10-induced CD19^high^ B lymphocytes

**DOI:** 10.1186/s40425-019-0744-4

**Published:** 2019-11-21

**Authors:** Yu-Li Chen, Han-Wei Lin, Chung-Liang Chien, Yen-Ling Lai, Wei-Zen Sun, Chi-An Chen, Wen-Fang Cheng

**Affiliations:** 1 Department of Obstetrics and Gynecology, College of Medicine, National Taiwan University, Taipei, 100 Taiwan; 2 Graduate Institute of Oncology, National Taiwan University, Taipei, Taiwan; 3 Graduate Institute of Anatomy and Cell Biology, National Taiwan University, Taipei, Taiwan; 4 Department of Anesthesiology, National Taiwan University, Taipei, Taiwan; 5 Graduate Institute of Clinical Medicine, College of Medicine, National Taiwan University, Taipei, Taiwan

**Keywords:** Cytokine, BTLA, Prognosis, BTLA^+^CD19^hi^ B lymphocyte, Cancer immunotherapy

## Abstract

**Background:**

The standard treatment for epithelial ovarian carcinoma (EOC) is surgery followed by platinum/paclitaxel-based chemotherapy, but the overall survival rate is poor. The purpose of this study was to investigate the therapeutic potential of chemotherapy combined with inhibition of B and T lymphocyte attenuator (BTLA) for clinical use to treat EOC.

**Methods:**

Initially, we evaluated the potential application of chemotherapy combined with anti-BTLA antibody in an animal model. We then analyzed the distribution and regulation of BTLA expression on immunocytes in vitro. Finally, we examined the correlation between BTLA expression levels in cancerous tissues and prognosis in 254 EOC cases.

**Results:**

The combination of chemotherapy and anti-BTLA antibody for inhibiting BTLA significantly reduced peritoneal tumor volume and extended survival in tumor-bearing mice. In addition, BTLA could be identified mostly on B lymphocytes, especially on CD19^hi^ B cells, rather than on T lymphocytes and natural killer cells. Under regulation of interleukins 6 and 10, more BTLA^+^CD19^hi^ B lymphocytes could be induced through AKT and STAT3 signaling pathways. Detectable BTLA expression in ovarian cancerous tissues was associated with worse disease-free and overall survivals of EOC patients.

**Conclusions:**

BTLA detected in cancerous tissues can predict poor outcome of EOC patients. Inhibition of BTLA combined with chemotherapy can elevate immune activation and generate potent anti-tumor effects. Thus, the combination of chemotherapy and anti-BTLA antibody may hold potential clinical application for the treatment of EOC patients.

**Trial registration:**

The Trial Registration Number was NCT00854399.

## Background

Tumor-associated antigens can be immunogenic at some cancer sites in early neoplasia, including epithelial ovarian carcinomas (EOCs) [1–3]. However, the neoplastic cells eventually become uncontrolled by the immune system after tumor immunogenicity editing in three phases: elimination, equilibrium, and escape [1]. In the elimination phase, the cytotoxicity generated by antigen-specific T cells can destroy cancers [1]. However, the occasional cancer cell variant is not eradicated in the elimination phase and may enter the equilibrium phase, in which immunologic mechanisms prevent its outgrowth. Because of immune selection pressure on genetically unstable tumor cells in equilibrium, these tumor cells may enter the escape phase, in which immunity no longer inhibits their outgrowth.

One mechanism for tumor growth promotion in the escape phase is immune checkpoints [4]. Immune checkpoints include a large number of inhibitory pathways hardwired into the immune system that are essential for maintaining self-tolerance and modulating immune response duration and amplitude to minimize tissue damage. Tumors can activate certain immune checkpoint pathways as a mechanism of immune resistance, particularly against the cytotoxic effects of antigen-specific T cells. Ligand-receptor interactions induce the suppressive activities of immune checkpoints, which can be blocked by antibodies (Abs) to enhance endogenous anti-tumor effects [5]. Among the immune checkpoints, cytotoxic T-lymphocyte-associated antigen 4 (CTLA4) and programmed cell death protein 1 (PD-1) are well-known because of promising clinical applications of the respective monoclonal Abs against them [6–8].

In addition to CTLA4 and PD1, B and T lymphocyte attenuator (BTLA) is an immune checkpoint involved in suppressing immune responses [5]. BTLA includes two immunoreceptor tyrosine-based inhibitory motifs in its cytoplasmic region [9] and can be identified on various immunocytes such as T and B lymphocytes, macrophages, dendritic cells, and nature killer (NK) cells [10]. Increasing proliferation can be observed in BTLA^−/−^ lymphocytes [9, 11]. BTLA plays inhibitory roles in multiple disease models including experimental encephalomyelitis, colitis, and major histocompatibility complex-mismatched cardiac allograft by modulating T cell responses in BTLA^−/−^ mice [9, 12, 13]. In addition, BTLA can attenuate B cell function by targeting the phosphorylation of SYK, B cell linker protein, phospholipase C-γ 2, and NF-κB [14], and exhibit inhibitory function to prevent nature killer T (NKT) cell-mediated hepatitis [15].

EOCs are the most lethal gynecologic malignancy [16]. The standard treatment is surgery followed by platinum/paclitaxel-based chemotherapy, but the overall survival (OS) rate is around 35% [17, 18]. Identification of accurate biomarkers is crucial for prognosis and for finding therapeutic targets in EOC. As noted, EOCs are immunogenic [2, 3], and BTLA exerts inhibitory influences on several immunocytes [9, 12–15]. Therefore, we first evaluated the potential application of chemotherapy combined with anti-BTLA Ab in an animal model. We then analyzed the distribution and regulation of BTLA expression on immunocytes in vitro. Finally, the correlation between expression levels of BTLA in cancerous tissues and the prognosis of EOC patients were examined.

## Methods

### Patients and specimens

A total of 254 women diagnosed with EOC who underwent staging or debulking surgery were enrolled. Cancerous tissue specimens were collected during surgery, frozen in liquid nitrogen, and stored at − 70 °C until analysis [19].

The clinical records of these patients were prospectively reviewed to obtain medical parameters such as age, operative findings, pathological findings, disease relapse, and prognosis. We defined disease characteristics according to the system of International Federation of Gynecology and Obstetrics [20]. Stage I and II diseases were considered early stage and stage III and IV as advanced. The maximal residual tumor size after each operation was recorded and divided into two groups, ≤1 cm and >  1 cm. Except for women with stage IA and grade I tumors, all patients received three to six courses of adjuvant platinum-based chemotherapy.

After completion of the primary treatment, regular follow-up was arranged every 3 months for 3 years, and every 6 months thereafter. Magnetic resonance imaging or computerized tomography was arranged for suspected disease relapse. Recurrence was considered when tumor marker (CA125) levels were ≥ 2 times the upper normal limit in two consecutive tests with 2-week intervals, findings of imaging studies and aspiration cytology were abnormal, or there was a biopsy-proven disease. The period of time from completion of the primary treatment until the date of confirmed relapse, progression, or last follow-up was calculated as disease-free survival (DFS). The time from diagnosis until the date of disease-related death or last visit was defined as OS [19].

### Extraction of RNA in ovarian cancer tissues and reverse-transcription polymerase chain reaction (RT-PCR)

Total RNA from ovarian cancer tissues was isolated for cDNA synthesis using TRIzol reagent (Invitrogen) following the manufacturer’s instructions. The samples were subsequently passed over a Qiagen RNeasy column (Qiagen) to remove small fragments. Then, total mRNA was reverse-transcribed to cDNA by a Moloney murine leukemia virus reverse transcriptase kit (Invitrogen).

BTLA is an immune-regulatory receptor and its ligand is the herpesvirus entry mediator (HVEM). BTLA (receptor)-HVEM (ligand) interaction could generate inhibitory effects on immune response to result in immune tolerance [21]. Thus, the roles of BTLA and HVEM on ovarian cancer patients were investigated by analyzing their expressions in the cancerous tissues.

To detect the RNA expression of BTLA and HVEM in the ovarian cancerous tissues, RT-PCR with primers specific for BTLA, HVEM, and GAPDH were applied for 30 cycles. The sequences of PCR primers were as follows: BTLA sense, 5′-GTCATACCGCTGTTCTGCAA − 3 and anti-sense, 5′-TTGAGTTCGGTCCAATGACA-3′; and HVEM sense, 5′-AGTGTCTGCAGTGCCAAATG-3′ and anti-sense, 5′-TCACCTTCTGCCTCCTGTCT-3′. The sense primer ACCCAGAAGACTGTGGATGG and anti-sense primer TGCTGTAGCCAAATTCGTTG were used to detect GAPDH. The amplification products were separated by 1% agarose gel electrophoresis and visualized after staining with ethidium bromide.

### Quantitative real-time RT-PCR (qPCR)

BTLA, HVEM, and β-actin RNA were reverse-transcribed to cDNA and then analyzed in a LightCycler Real-Time detection system (Roche Diagnostics): BTLA (Hs00699198_m1), HVEM (Hs00998604_m1), and β-actin (Hs03023880_g1) by TaqMan® gene expression assays. Relative expression levels were presented as the 2^−ΔΔCt^ method using β-actin as internal control [22]. The quantitative data were calculated with the numbers of cycles for amplification-generated fluorescence to reach a specific detection threshold (Ct value). In this study, a cycle number > 40 was defined as non-detectable. The expression levels of CTLA-4 (Hs00175480_m1), PD-1(Hs01550088_m1), and programmed death-ligand 1 (PD-L1) (Hs00204257_m1) were also analyzed.

### Mice

Female C57BL/6 J mice age 6 to 8 weeks were purchased from National Taiwan University and bred in the animal facility of the National Taiwan University Hospital. All animal procedures were performed in accordance with approved protocols.

### Cell line

WF-3/Luc tumor cells for this ascitogenic animal model were generated from WF-3 tumor cells as described previously [23]. These cells were maintained in RPMI-1640, supplemented with 10% (volume/volume) fetal bovine serum, 50 U/mL penicillin/streptomycin, 2 mM L-glutamine, 1 mM sodium pyruvate, 2 mM non-essential amino acids, and 0.4 mg/mL G418 at 37 °C with 5% carbon dioxide.

In this animal model, two time points were indicated, day 14 (14 days after tumor challenge) as early disease and day 35 (35 days after tumor challenge) as advanced disease. Analysis of immune components at these two time points can display the alterations of host immunities in the tumor progression [24]. In addition, the tumor cells can intraperitoneally spread with ascites formation. Apart from tumor cells, different kinds of tumor-associated cells (TACs) including lymphocytes and regulatory elements such as cytokines could be detected from the tumor-related ascites. The malignant ascites could be regarded as part of the tumor microenvironment (TME) to reflect the association between host immunity and tumor cells in this TME [25, 26].

#### Determination of drug doses

To determine the daily drug doses for all experiments, weight loss and clinical scores were employed as two endpoints. Clinical scores were based on mice activity, appearance and body condition as described previously [27]. The starting doses were selected by literature review, taking a dose that was safe to administer. Doses were escalated at increments of 25% of starting doses. When any mice met the endpoint of either reached a clinical score > 2 or >  15% weight loss within 2 weeks, dose escalation was ceased and the prior dose was set as the determined drug doses. For anti-BTLA Ab, half of the determined dose was additionally selected to investigate dose relationship between anti-BTLA Ab-contained treatment and anti-tumor effects.

### In vivo tumor treatment

The therapeutic agents, including paclitaxel, cisplatin, bevacizumab, and olaparib (all from Sigma-Aldrich), diluted with DMSO, was intraperitoneally administered to mice. Anti-BTLA Ab (clone 6A6, Bio X cell) [28], anti-PD-1 Ab (clone RMP1–14, Bio X cell) [29], anti-PD-L1 Ab (clone 10F.9G2, Bio X cell) [30], anti-CD19 Ab (clone 1D3, Bio X cell) [31], LY294002 (Selleck Chemicals) [32], and BP-1-102 (Selleck Chemicals) [33] were also used for the following experiments.

Briefly, C57BL/6 J mice (10 mice per group) were intraperitoneally challenged with 1 × 10^5^ WF-3/Luc tumor cells on day 0. On day 3, paclitaxel (6 mg/kg, intraperitoneal use) and/or several agents, including anti-BTLA Ab (10 or 20 μg/mouse, intraperitoneal use), anti-PD-1 Ab (30 μg/mouse, intraperitoneal use), anti-PD-L1 Ab (30 μg/mouse, intraperitoneal use), anti-CD19 Ab (30 μg/mouse, intraperitoneal use), LY294002 (800 μg/mouse, intraperitoneal use), or BP-1-102 (40 μg/mouse, oral use), were administered daily until the day of euthanasia. In addition to paclitaxel, other therapeutic agents, including cisplatin (1 mg/kg, intraperitoneal use), bevacizumab (2 mg/kg, intraperitoneal use), or olaparib (5 mg/kg, intraperitoneal use) and/or anti-BTLA Ab (20 μg/mouse, intraperitoneal use) were daily applied to evaluate the anti-tumor effects since day 3. Mice were sacrificed on the indicated day for immunologic profiling assays, and the remaining animals (5 in each group) were kept until 100 days after tumor challenge or death for the OS analysis. Therapy was discontinued on day 100. Then, the surviving mice were subcutaneously re-challenged with 1 × 10^5^ WF-3/Luc tumor cells. Bioluminescence images of tumor growth were detected twice a week using an IVIS Imaging System Series 200 (Xenogen) [23].

#### Preparation of splenocytes, tumor-infiltrating lymphocytes (TILs), supernatants and TACs of cancer-related ascites

The splenocytes, TILs, supernatants and TACs of ascites from WF3/Luc tumor model were obtained on the indicated day [24, 29]. The splenocytes and TILs were then used directly or stored at − 135 °C. The ascites specimens were separated into supernatants and cellular components by centrifugation at 2000 rpm for 5 min. The supernatants and the cells were stored at − 20 °C and − 135 °C, respectively. The splenocytes, TILs, and TACs were cryopreserved with freezing media (FBS containing medium + 10% DMSO).

#### Surface marker staining and flow cytometry of splenocytes, TILs and TACs

The murine splenocytes, TILs and TACs were stained with fluorescein isothiocyanate (FITC)-conjugated CD3 (Biolegend), allophycocyanin (APC)-conjugated CD4 (Biolegend), phycoerythrin (PE)/Cy5.5-conjugated CD8 (Biolegend), PE-conjugated NK1.1 (Biolegend), PE/Cy5.5-conjugated CD19 (Biolegend), APC-conjugated BTLA (CD272) (Biolegend), or PE-conjugated CD223 (eBioscience) for different experiments. Flow cytometric analyses were performed using a BD FACSCalibur flow cytometer (BD Bioscience) with CELLQuest software [23, 24].

### Tumoricidal activity of splenocytes from tumor-bearing mice receiving chemotherapy treated with or without anti-BTLA Ab in vitro

Splenocytes of tumor-bearing mice treated with daily intraperitoneal paclitaxel 6 mg/kg for 14 days were harvested as described earlier. These splenocytes were first incubated in vitro with/without anti-BTLA Ab (10 or 20 μg/mL) for 1 h and then co-cultured with the irradiated WF-3/Luc tumor cells at various ratios (WF-3/Luc:splenocyte = 1:100, 1:50, 1:10, and WF-3/Luc only) in a 96-well plate (1 × 10^4^ cells/well) for 48 h. Irradiated WF-3/Luc tumor cells treated only with PBS or anti-BTLA Ab (10 or 20 μg/mL) were considered as control. The luciferase activities of tumor growth were measured using the IVIS Imaging System Series 200 (Xenogen), as described previously [23].

### Enzyme-linked immunosorbent assays (ELISA) of cytokines in ascites of tumor-bearing mice

Direct ELISAs of murine interleukin (IL)-6, − 10, − 12, transforming growth factor-beta (TGF-β), Tumor necrosis factor-alpha (TNF-α), and interferon-gamma (IFN-γ) (e-Bioscience) in the supernatants of ascites were performed based on the manufacturer’s instructions [24].

### Sorting of B lymphocyte by flow cytometry

Splenocytes were first obtained as described earlier and then stained with FITC-conjugated anti-mouse CD3 (Biolegend) and PE/Cy5.5-conjugated anti-mouse CD19 (Biolegend). The CD3^−^CD19^+^ cells (B lymphocytes) were sorted for further analysis on FACSAriaIII (BD Bioscience) by the Flow Cytometric Analyzing and Sorting Core Facility at National Taiwan University Hospital.

### The effect of IL-6, IL-10, or TGF-β on BTLA^+^CD19^high (hi)^ B lymphocytes

The B lymphocytes were first sorted as described. PBS, recombinant mouse IL-6 (20 ng/mL), IL-10 (20 ng/mL), or TGF-β (10 ng/mL) (PeproTech) was loaded with these collected B lymphocytes for 24 h. Then, the percentage of BTLA^+^CD19^hi^ B lymphocytes was analyzed by flow cytometry.

### Western blot and flow cytometric analyses for the signaling pathway of BTLA expression

For the signal transduction pathways of IL-6 and IL-10 in B lymphocytes, western immunoblotting was performed [23]. Briefly, the sorted B cells (1 × 10^6^/well) were treated with serum-free media and seeded in a 24-well plate for 6 h. Then B cells were treated with PBS, IL-6 (10 and 20 ng/mL), and IL-10 (10 and 20 ng/mL) and harvested after 15 min of incubation. The protein extracts were quantified with a BCA Protein Assay Kit (Pierce). Then, 20 μg of each cell lysate was resolved by SDS/PAGE (10% gel), transferred onto a PVDF/nylon membrane (Millipore), and probed with Abs specific to phospho-STAT3, phospho-AKT, phospho-ERK, total STAT3, total AKT, total ERK, α-tubulin, and GAPDH (Genetex). The membrane was then probed with HRP-conjugated secondary Ab. The specific bands were visualized using an ECL® Western blotting system (GE Healthcare).

To analyze the efficiency of blockades of possible pathways in B lymphocytes, the anti-BTLA Ab (20 μg/mL), AKT (LY294002, 25 μM), STAT3 (BP-1-102, 2 μM), or ERK (PD98059, 10 μM) inhibitor was first incubated with sorted B cells for 1 h. Then, these cells were treated with PBS, IL-6 (20 ng/mL), IL-10 (20 ng/mL), or TGF-β (10 ng/mL) for the following 24 h. These B cells were analyzed to detect the phosphorylation status of STAT3, AKT, and ERK by western immunoblotting and to evaluate the percentages of BTLA^+^CD19^hi^ B lymphocytes by flow cytometry.

### Statistical analysis

All statistical analyses were performed with SPSS for Windows version 15.0 (SPSS Inc., Chicago, IL). The clinicopathological characteristics between BTLA non-detectable and detectable groups were analyzed using the Chi-square test for dichotomized variables and the Mann-Whitney U test for continuous variables. Risk analysis of cancer recurrence and disease-related death was completed with the Cox regression model for hazard ratio (HR) and 95% confidence interval (CI). Spearman’s rank correlation was employed to evaluate the relationship between two immune checkpoints. The correlation coefficient, R value ≥0.4 was considered as correlated.

The in vivo and in vitro data were shown as mean ± SE (standard error), which represented at least two different experiments. The results of luminescence, ELISA, and flow cytometry were evaluated with the Kruskal-Wallis test. In the survival experiments, the event time distributions were analyzed by Kaplan-Meier method and log rank test. A *p* < 0.05 was defined as statistically significant.

## Results

### Chemotherapy combined with BTLA inhibition could generate more potent anti-tumor effects

**Fig. 1:**
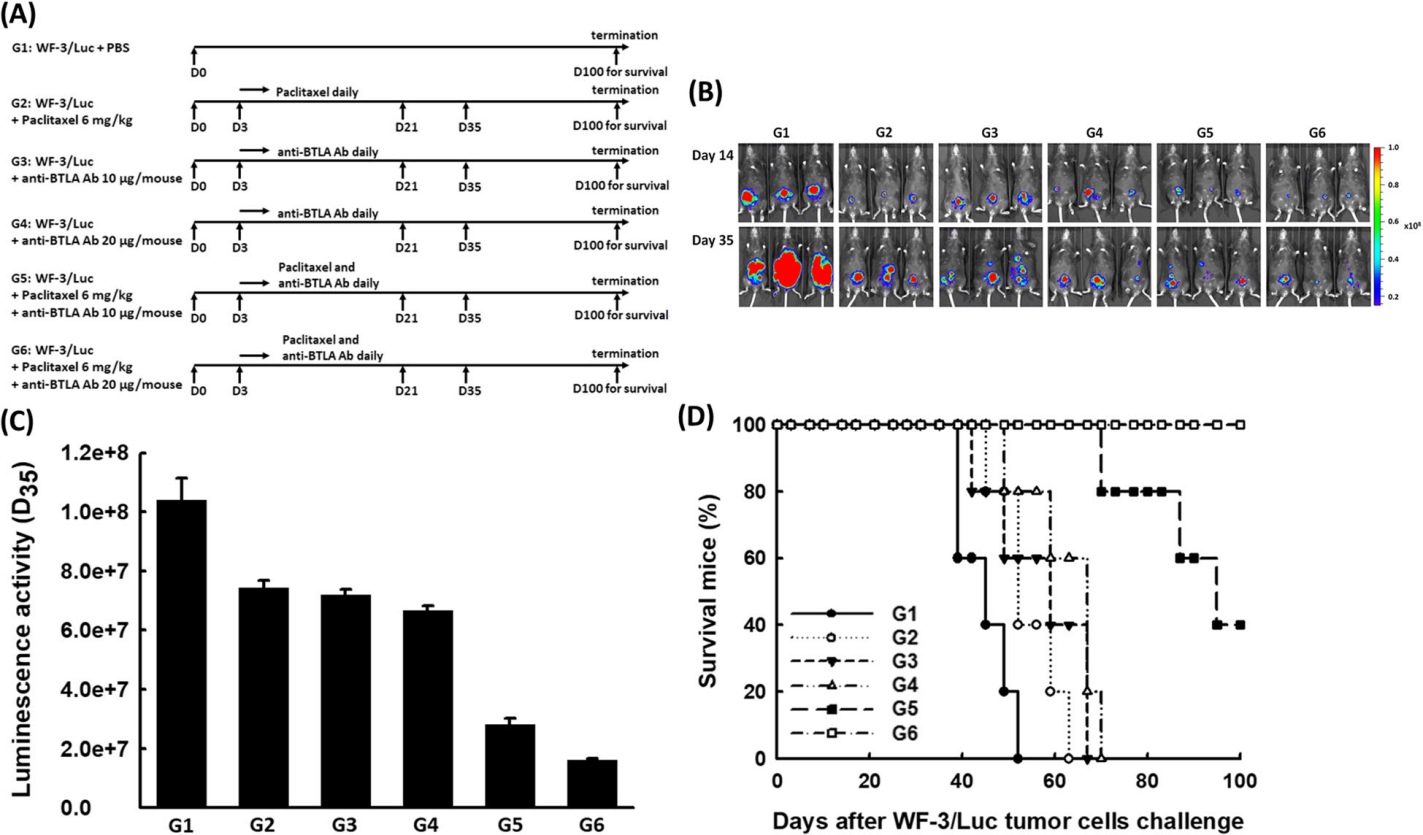
Chemotherapy combined with anti-BTLA Ab significantly reduced peritoneal tumor volumes and extended survival of tumor-bearing mice. **a** Diagrammatic representation of different treatment protocols using paclitaxel and/or anti-BTLA Ab. *Note:* G1: PBS only; G2: paclitaxel 6 mg/kg; G3: anti-BTLA Ab 10 μg/mouse; G4: anti-BTLA Ab 20 μg/mouse; G5: paclitaxel 6 mg/kg and anti-BTLA Ab 10 μg/mouse; G6: paclitaxel 6 mg/kg and anti-BTLA Ab 20 μg/mouse. **b** Representative luminescence images of mice in various groups using the IVIS system on the indicated days after tumor challenge. (5 mice in each group) **c** Luminal analyses of tumor volumes in tumor-bearing mice with various regimens. Mice treated with paclitaxel and anti-BTLA Ab 20 μg/mouse exhibited the least luminescence (*p* < 0.001, Kruskal-Wallis test). (5 mice in each group). **d** Survival analysis of mice in the various groups. All mice treated with paclitaxel and anti-BTLA Ab 20 μg/mouse and 40% of mice treated with paclitaxel and anti-BTLA Ab 10 μg/mouse were alive 100 days after the WF-3/Luc tumor challenge. However, none of the mice in the other groups survived more than 70 days of tumor challenge (*p* < 0.001, log-rank test). (5 mice in each group)

Chemotherapy plays an important role on the treatment of EOCs. Integrating potential targets, including immune checkpoint blockades for enhancing the anti-tumor effects of chemotherapeutic agents is an emerging issue. Accordingly, to preclinically explore whether the combination of chemotherapy and BTLA inhibition has a synergistic impact on generating more potent anti-tumor effects, the mAb 6A6 was used for in vivo BTLA inhibition via variRous treatment protocols (Fig. 1a). The luciferase activities of WF-3/Luc tumors in mice with various regimens detected by the IVIS system are shown in Fig. 1b. The luciferase activities of mice treated with anti-BTLA Ab 10 μg/mouse (G3), or anti-BTLA Ab 20 μg/mouse (G4) alone were lower than those of PBS-treated group (G1) (*p* = 0.004, Kruskal-Wallis test) but similar to those of paclitaxel-treated group (G2) (*p* = 0.085, Kruskal-Wallis test) (Fig. 1c). Therefore, the anti-tumor effects of combination therapy with different mechanisms were further explored. The mice undergoing chemotherapy combined with anti-BTLA Ab 20 μg/mouse (G6: paclitaxel 6 mg/kg and anti-BTLA Ab 20 μg/mouse, 1.63 ± 0.04 × 10^7^) exhibited the least luminescence 35 days after tumor inoculation (G1: PBS-treated group, 1.04 ± 0.07 × 10^8^; G2: paclitaxel 6 mg/kg, 7.44 ± 0.25 × 10^7^; G3: anti-BTLA Ab 10 μg/mouse, 7.21 ± 0.18 × 10^7^; G4: anti-BTLA Ab 20 μg/mouse, 6.67 ± 0.17 × 10^7^; G5: paclitaxel 6 mg/kg and anti-BTLA Ab 10 μg/mouse, 2.82 ± 0.19 × 10^7^; *p* < 0.001, Kruskal-Wallis test, Fig. 1c).

None of the mice treated with paclitaxel or anti-BTLA alone could survive 70 days after tumor challenge. All mice treated with paclitaxel and anti-BTLA Ab 20 μg/mouse and 40% of mice treated with paclitaxel and anti-BTLA Ab 10 μg/mouse were still alive even 100 days after the WF-3/Luc tumor challenge (*p* < 0.001, log-rank test, Fig. 1d). Furthermore, mice treated with paclitaxel and anti-BTLA Ab 20 μg/mouse were re-challenged with WF-3/Luc tumor cells 100 days after the first tumor challenge. By the IVIS system, the subcutaneous re-challenged tumors of mice could be detected (Additional file 1: Figure S1).

In addition to paclitaxel, mice undergoing cisplatin 1 mg/kg (*p* = 0.02, log-rank test, Additional file 2: Figure S2A), bevacizumab 2 mg/kg (*p* < 0.001, log-rank test, Additional file 2: Figure S2B), or olaparib 5 mg/kg (*p* = 0.01, log-rank test, Additional file 2: Figure S2C) combined with anti-BTLA Ab 20 μg/mouse had longer survival intervals than those treated with respective agent alone. Sixty percent of mice treated with bevacizumab and anti-BTLA Ab (Additional file 2: Figure S2B) and 40% of mice treated with olaparib and anti-BTLA Ab (Additional file 2: Figure S2C) were still alive 100 days after tumor challenge.

The survivals of mice treated with paclitaxel 6 mg/kg, anti-BTLA Ab 20 μg/mouse, anti-PD-1 Ab 30 μg/mouse, or anti-PD-L1 Ab 30 μg/mouse alone did not show difference (*p* = 0.39, log-rank test, Additional file 3: Figure S3A). Sixty percent of mice treated with paclitaxel and anti-PD-L1 Ab and 80% of mice treated with paclitaxel and anti-PD-1 Ab were alive 100 days after tumor challenge. All of the mice treated with paclitaxel and anti-BTLA Ab, paclitaxel, anti-PD-1 Ab and anti-BTLA Ab, or paclitaxel, anti-PD-L1 Ab and anti-BTLA Ab were alive 100 days after tumor challenge (Additional file 3: Figure S3B).

Therefore, chemotherapy combined with anti-BTLA Ab can generate more potent anti-tumor effects than chemotherapy or anti-BTLA Ab alone. Paclitaxel combined with anti-BTLA Ab displayed the highest survival rate.

### Tumor-bearing mouse host immunity tends to be activated with anti-tumor effects under combination treatment with chemotherapy and anti-BTLA Ab

We further evaluated if the immune profiles could correlate with the anti-tumor effects of mice treated with different strategies. The immunologic alternations including the activated T lymphocytes in splenocytes and TACs of ascites, in vitro tumor killing abilities of splenocytes and the expression levels of various pro- and anti-inflammatory cytokines in ascites were detected. CD223 was used as the activating marker of T lymphocytes [23, 24].

**Fig. 2:**
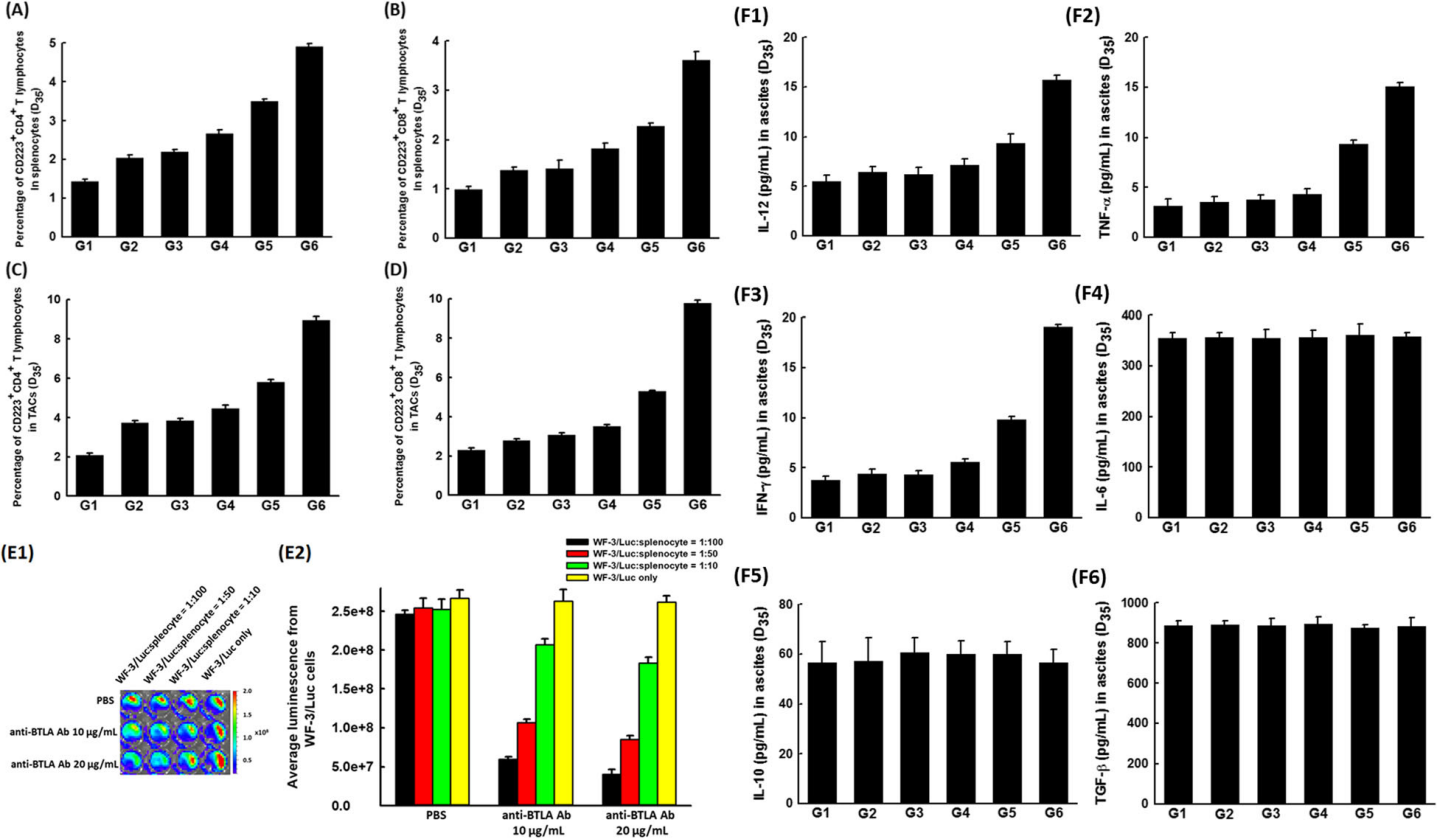
Immunologic alterations in tumor-bearing mice treated with chemotherapy and/or anti-BTLA Ab. **a** The percentages of CD223 expression of CD4^+^ T lymphocytes in splenocytes of various therapeutic groups. The percentage of CD223^+^CD4^+^ T lymphocytes in splenocytes was highest in paclitaxel combined with anti-BTLA Ab 20 μg/mouse group (*p* = 0.001, Kruskal-Wallis test). (5 mice in each group) **b** The percentages of CD223 expression of CD8^+^ T lymphocytes in splenocytes of various therapeutic groups. The percentage of CD223^+^CD8^+^ T lymphocytes in splenocytes was also highest with paclitaxel combined with anti-BTLA Ab 20 μg/mouse (*p* = 0.001, Kruskal-Wallis test). (5 mice in each group) **c** The percentages of CD223 expression of CD4^+^ T lymphocytes in TACs of ascites of various therapeutic groups. The percentage of CD223^+^CD4^+^ T lymphocytes in TACs of ascites was highest with paclitaxel and anti-BTLA Ab 20 μg/mouse (*p* = 0.001, Kruskal-Wallis test). (5 mice in each group) **d** The percentages of CD223 expression of CD8^+^ T lymphocytes in TACs of ascites of various therapeutic groups. The percentage of CD223^+^CD8^+^ T lymphocytes in TACs of ascites was also highest with paclitaxel combined with anti-BTLA Ab 20 μg/mouse (*p* = 0.001, Kruskal-Wallis test). (5 mice in each group) **e** Tumoricidal activity of splenocytes of tumor-bearing mice receiving chemotherapy treated with/without anti-BTLA Ab in vitro. **e1** Representative luminescence figures of the in vitro tumor killing abilities of splenocytes by the IVIS system. (5 mice in each group) **e2** Quantification of luminescence of in vitro tumor killing abilities of splenocytes by the IVIS system. Compared with the luminescence of WF-3/Luc cells co-cultured with splenocytes without anti-BTLA Ab, less luminal activity was detected in WF-3/Luc cells co-cultured with splenocytes receiving in vitro anti-BTLA Ab (*p* = 0.021 for WF-3/Luc:splenocyte = 1:100; *p* = 0.027 for WF-3/Luc:splenocyte = 1:50; and *p* = 0.039 for WF-3/Luc:splenocyte = 1:10, Kruskal-Wallis test). The splenocytes treated with anti-BTLA Ab could generate higher tumor killing activities than those without anti-BTLA Ab. (5 mice in each group) **f** Bar figures of concentrations of various cytokines in ascites of various groups. *Note*: F1: IL-12; F2: TNF-α; F3: IFN-γ; F4: IL-6; F5: IL-10; and F6: TGF-β. The pro-inflammatory cytokines such as IL-12 (*p* = 0.002), TNF-α (*p* = 0.002), and IFN-γ (*p* = 0.001) were highest with the chemotherapy combined with anti-BTLA Ab 20 μg/mouse. The concentrations of ant-inflammatory cytokines such as IL-6 (*p* = 0.83), IL-10 (*p* = 0.85), and TGF-β (*p* = 0.84) did not show differences among the various groups (all statistical analyses by Kruskal-Wallis test). (5 mice in each group)

Compared with the other groups, the percentages of CD223^+^CD4^+^ (G6: 4.91 ± 0.08%; *p* = 0.001, Kruskal-Wallis test, Fig. 2a) and CD223^+^CD8^+^ T (G6: 3.61 ± 0.18%; *p* = 0.001, Kruskal-Wallis test, Fig. 2b) lymphocytes in splenocytes was highest in chemotherapy combined with anti-BTLA Ab 20 μg/mouse group. In addition, similar phenomena were identified in the TACs of ascites. The percentages of CD223^+^CD4^+^ (G6: 8.95 ± 0.18%; *p* = 0.001, Kruskal-Wallis test, Fig. 2c) and CD223^+^CD8^+^ (G6: 9.77 ± 0.15%; *p* = 0.001, Kruskal-Wallis test, Fig. 2d) T lymphocytes were also highest in chemotherapy combined with anti-BTLA Ab 20 μg/mouse group.

For luminescence evaluation of in vitro tumor killing abilities of splenocytes, splenocytes harvested from the tumor-bearing mice undergoing chemotherapy were incubated with/without anti-BTLA Ab and then co-cultured with the irradiated WF-3/Luc tumor cells at various ratios (WF-3/Luc:splenocyte = 1:100, 1:50, 1:10, and WF-3/Luc only) (Fig. 2e1). WF-3/Luc cells co-cultured with splenocytes receiving anti-BTLA Ab (anti-BTLA Ab 10 μg/mL group: 2.07 ± 0.08 × 10^8^; anti-BTLA Ab 20 μg/mL group: 1.83 ± 0.07 × 10^8^) showed less luminal activity compared with those co-cultured with splenocytes without anti-BTLA Ab (2.53 ± 0.13 × 10^8^) (WF-3/Luc:splenocyte = 1:10; *p* = 0.039, Kruskal-Wallis test, Fig. 2e2).

In addition, the pro-inflammatory cytokines including IL-12 (G6: 15.69 ± 0.51 pg/mL; *p* = 0.002, Kruskal-Wallis test, Fig. 2f1), TNF-α (G6: 15.06 ± 0.38 pg/mL; *p* = 0.002, Kruskal-Wallis test, Fig. 2f2), and IFN-γ (G6: 19.07 ± 0.26 pg/mL; *p* = 0.001, Kruskal-Wallis test, Fig. 2f3) were higher in ascites of tumor-bearing mice treated with paclitaxel combined with anti-BTLA Ab 20 μg/mouse than those of other groups. However, the concentrations of anti-inflammatory cytokines such as IL-6 (*p* = 0.83, Kruskal-Wallis test, Fig. 2f4), IL-10 (*p* = 0.85, Kruskal-Wallis test, Fig. 2f5), or TGF-β (*p* = 0.84, Kruskal-Wallis test, Fig. 2f6) showed no significant difference among the various groups.

These results showed that inhibition of BTLA could enhance host anti-tumor immunity and anti-tumor effects when combined with chemotherapy.

### IL-6 and IL-10 could enhance the quantity of BTLA^+^CD19^hi^ B lymphocytes through AKT and STAT3 signaling pathways

**Fig. 3:**
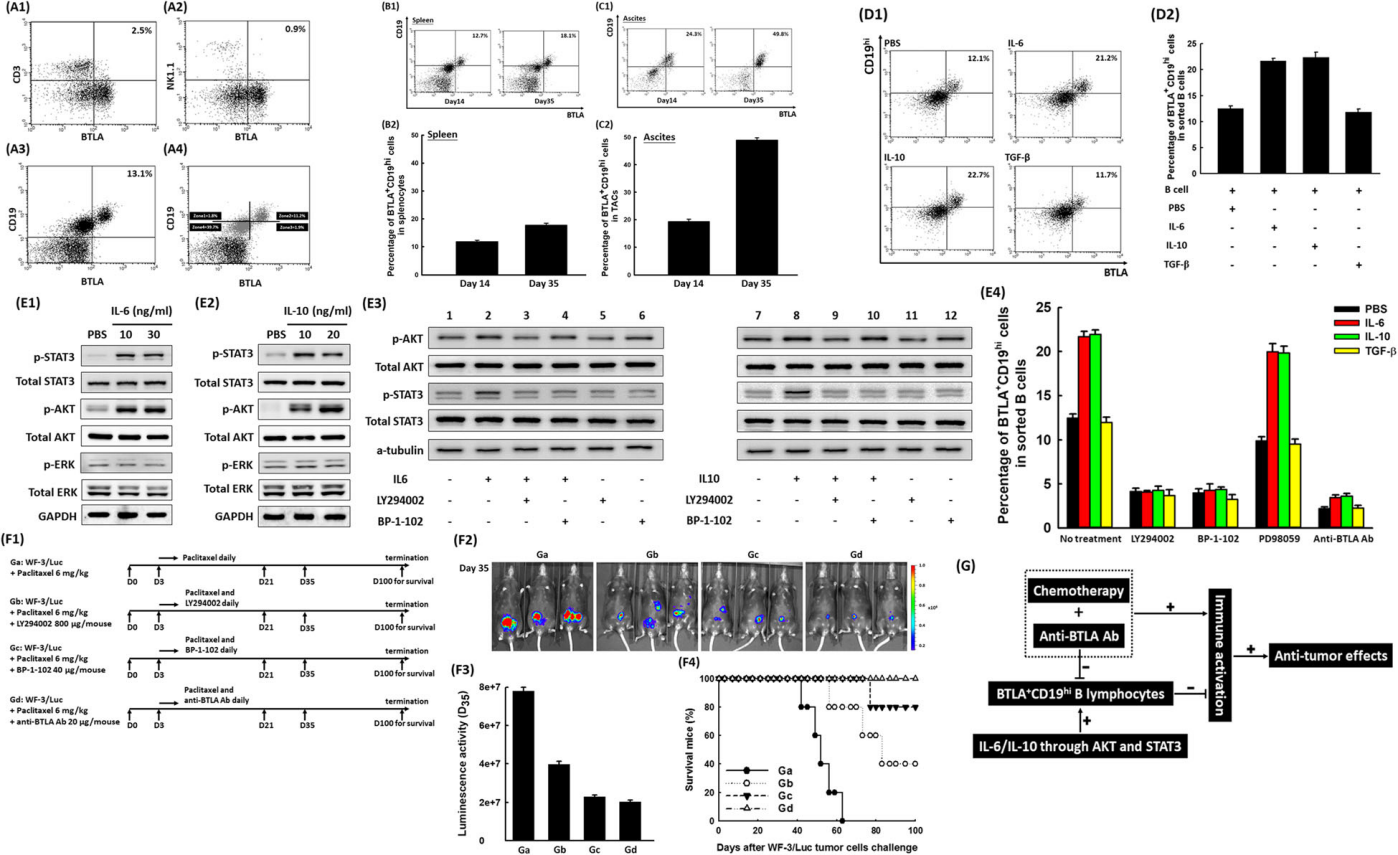
IL-6 and IL-10 could induce more BTLA^+^CD19^hi^ B lymphocytes through the AKT and STAT3 signaling pathways. **a** Representative figures of flow cytometric analyses of the expression of BTLA on various kinds of immunocytes of splenocytes. *Note:* A1: T lymphocytes; A2: NK cells; A3: B lymphocytes; A4: subgroups of B lymphocytes (zone 1: BTLA^−^CD19^hi^; zone 2: BTLA^+^CD19^hi^; zone 3: BTLA^+^CD19^low(lo)^; zone 4: BTLA^+^CD19^lo^). B lymphocytes, especially CD19^hi^ B lymphocytes, had higher percentages expressing the BTLA molecule. (5 mice in this analysis) **b** Kinetic alterations in BTLA^+^CD19^hi^ B lymphocytes in splenocytes of tumor-bearing mice after different days of tumor challenge. **b1** Representative flow cytometric figures of percentages of BTLA^+^CD19^hi^ B lymphocytes in splenocytes on indicated days. (5 mice in each group) **b2** Bar figures exhibited the percentages of BTLA^+^CD19^hi^ B lymphocytes in splenocytes on day 14 or day 35 after tumor challenge. The percentages of BTLA^+^CD19^hi^ B lymphocytes were higher on day 35 (17.74 ± 0.71%) than on day 14 (11.76 ± 0.52%) (*p* = 0.009, Kruskal-Wallis test). (5 mice in each group) **c** Kinetic alterations in BTLA^+^CD19^hi^ B lymphocytes in TACs of ascites from tumor-bearing mice after different days of tumor challenge. **c1** Representative flow cytometric figures of BTLA^+^CD19^hi^ B lymphocytes in TACs at indicated intervals. (5 mice in each group) **c2** Bar figures of the percentages of BTLA^+^CD19^hi^ B lymphocytes in TACs on day 14 or day 35 after tumor challenge. The percentages of BTLA^+^CD19^hi^ B lymphocytes were higher on day 35 (48.94 ± 0.92%) than on day 14 (19.34 ± 0.88%) (*p* = 0.007, Kruskal-Wallis test). (5 mice in each group) **d** Alterations in the percentages of BTLA^+^CD19^hi^ B lymphocytes in sorted B lymphocytes treated with IL-6, IL-10, or TGF-β, analyzed by flow cytometry. **d1** Representative flow cytometric figures of the percentages of BTLA^+^CD19^hi^ B lymphocytes in sorted B cells. (5 mice in each group) **d2** Bar figures of the percentages of BTLA^+^CD19^hi^ B lymphocytes in sorted B cells treated with respective cytokines. The percentages of BTLA^+^CD19^hi^ B lymphocytes increased under treatment with IL-6 or IL-10 compared with TGF-β (*p* = 0.033, Kruskal-Wallis test). (5 mice in each group) **e** Various signaling molecules of sorted B lymphocytes treated with IL-6 and IL-10, detected by western blotting and flow cytometric analyses. **e1** IL-6 (10 or 20 ng/mL) could stimulate phosphorylation of STAT3 and AKT in sorted B lymphocytes. (5 mice in each group) **e2** Phosphorylation of STAT3 and AKT in sorted B cells also could be promoted by IL-10 (10 or 20 ng/mL). (5 mice in each group) **e3** The inhibition of p-AKT by LY294002 showed inhibition of p-STAT3 (Lanes 3 and 9). However, the inhibition of p-STAT3 by BP-1-102 did not block activation of p-AKT (Lanes 4 and 10). Therefore, AKT activation was upstream of STAT3 in the IL-6/IL-10 signaling pathway. (5 mice in each group) **e4** Percentages of BTLA^+^CD19^hi^ B lymphocytes in sorted B lymphocytes pretreated with respective Ab or specific inhibitor and then incubated with respective cytokine, analyzed by flow cytometry. The percentages of BTLA^+^CD19^hi^ B lymphocytes decreased when the B lymphocytes were pretreated with anti-BTLA Ab, LY294002 (AKT inhibitor), or BP-1-102 (STAT3 inhibitor) compared with PD98059 (ERK inhibitor). (5 mice in each group) **f** Anti-tumor effects of chemotherapy combined with various BTLA-related inhibitors. (F1) Diagrammatic representation of different treatment protocols using paclitaxel and various BTLA inhibitors. *Note:* Ga: paclitaxel 6 mg/kg; Gb: paclitaxel 6 mg/kg and LY294002 800 μg/mouse; Gc: paclitaxel 6 mg/kg and BP-1-102 40 μg/mouse; Gd: paclitaxel 6 mg/kg and anti-BTLA Ab 20 μg/mouse. (F2) Representative luminescence images of mice in various groups using the IVIS system on day 35 after tumor challenge. (5 mice in each group) (F3) Luminal analyses of tumor volumes in tumor-bearing mice with various regimens. Mice treated with paclitaxel and various BTLA-related inhibitors exhibited less luminescence than the paclitaxel-treated group (*p* < 0.001, Kruskal-Wallis test). Among mice receiving paclitaxel and various BTLA-related inhibitors, those receiving paclitaxel and anti-BTLA Ab 20 μg/mouse showed the least luciferase activity (*p* = 0.002, Kruskal-Wallis test). (5 mice in each group) (F4) Survival analysis of mice in various groups. Animals treated with chemotherapy and respective BTLA-related inhibitor lived longer than those treated only with paclitaxel (*p* < 0.001, log-rank test). All mice treated with paclitaxel and anti-BTLA Ab 20 μg/mouse, 80% of mice treated with paclitaxel and BP-1-102 40 μg/mouse, and 40% of animals treated with paclitaxel and LY294002 800 μg/mouse were alive 100 days after WF-3/Luc tumor challenge. (5 mice in each group) **g** Schematic diagram showing possible regulation and preclinical application of BTLA

To investigate the mechanism of regulating BTLA expression during tumor progression, the distribution of BTLA on immunocytes in mouse spleen was first analyzed by flow cytometry (Fig. 3a). As shown in Fig. 3a1-a3, BTLA can be expressed mostly on B lymphocytes rather than T lymphocytes and NK cells. When these B lymphocytes were further subclassified, BTLA was largely identified on the CD19^hi^ B lymphocytes (Fig. 3a4). Therefore, the BTLA^+^CD19^hi^ B lymphocytes were used to evaluate the regulation of BTLA expression.

Figure 3b1 and c1 give representative percentages of BTLA^+^CD19^hi^ B lymphocytes in splenocytes and TACs of ascites in tumor-bearing mice on day 14 (early disease) and day 35 (advanced disease), as determined by flow cytometry. The percentage of BTLA^+^CD19^hi^ B lymphocytes in splenocytes on day 35 (17.74 ± 0.71%) was higher than on day 14 (11.76 ± 0.52%) (*p* = 0.009, Kruskal-Wallis test, Fig. 3b2). The percentage of BTLA^+^CD19^hi^ B lymphocytes in TACs on day 35 (48.94 ± 0.92%) was also higher than on day 14 (19.34 ± 0.88%) (*p* = 0.007, Kruskal-Wallis test, Fig. 3c2). Similar alterations of the percentages of BTLA^+^CD19^hi^ B lymphocytes between day 14 (5.46 ± 0.58%) and day 35 (18.18 ± 0.65%) were also identified in TILs of the TME (*p* = 0.009, Kruskal-Wallis test, Additional file 4: Figure S4). The disparity in percentages of BTLA^+^CD19^hi^ B lymphocytes between early and advanced disease was noticeably larger in TACs of ascites than in splenocytes or TILs.

The concentrations of IL-6, IL-10, and TGF-β significantly increased in the ascites during tumor progression, as we showed in a previous study [24]. Therefore, the sorted B lymphocytes were treated with IL-6, IL-10, or TGF-β to evaluate impacts of these cytokines on the alterations of BTLA^+^CD19^hi^ B lymphocytes in vitro. The representative figures of percentages of BTLA^+^CD19^hi^ B lymphocytes in sorted B lymphocytes treated with IL-6, IL-10, or TGF-β by flow cytometry are shown in Fig. 3d1. The percentages of BTLA^+^CD19^hi^ B lymphocytes of B lymphocytes significantly increased under treatment with IL-6 (21.68 ± 0.48%) or IL-10 (22.43 ± 0.92%) compared with PBS (12.57 ± 0.53%) or TGF-β (11.92 ± 0.60%) (*p* = 0.033, Kruskal-Wallis test, Fig. 3d2).

We further elucidated the possible signaling molecules involved in the BTLA expression of B lymphocytes regulated by IL-6 and IL-10. As shown in Fig. 3e1 and e2, IL-6 or IL-10 could enhance phosphorylation of STAT3 and AKT molecules but not ERK in B lymphocytes. Inhibition of p-AKT by LY294002 could block activation of p-STAT3 (Lanes 3 and 9, Fig. 3e3); however, inhibition of p-STAT3 by BP-1-102 did not block p-AKT activation (Lanes 4 and 10). Therefore, AKT could regulate activation of STAT3 in the IL-6/IL-10 signaling pathway. The percentages of BTLA^+^CD19^hi^ B lymphocytes of IL-6- or IL-10-treated B lymphocytes significantly decreased when the B lymphocytes were pretreated with anti-BTLA Ab, LY294002 (AKT inhibitor), or BP-1-102 (STAT3 inhibitor) (Fig. 3e4).

Since anti-BTLA Ab, LY294002, and BP-1-102 had the ability to down-regulate the percentages of BTLA^+^CD19^hi^ B lymphocytes in vitro, the in vivo anti-tumor effects of these molecules were further investigated. Consequently, we evaluated the anti-tumor effects of chemotherapy combined with various BTLA-related inhibitors such as anti-BTLA Ab, LY294002, and BP-1-102 (Fig. 3f1). The luciferase activities of WF-3/Luc tumors in mice with various treatment protocols are shown in Fig. 3f2. Mice treated with paclitaxel and various BTLA-related inhibitors exhibited less luminescence than the paclitaxel-treated group (*p* < 0.001, Kruskal-Wallis test, Fig. 3f3). Among mice receiving paclitaxel and respective BTLA-related inhibitor, the paclitaxel and anti-BTLA Ab 20 μg/mouse group showed the lowest luciferase activities (2.04 ± 0.08 × 10^7^) (*p* = 0.002, Kruskal-Wallis test). The survival of mice treated with paclitaxel and respective BTLA-related inhibitor was longer than in the paclitaxel-treated group (*p* < 0.001, log-rank test, Fig. 3f4). All mice treated with paclitaxel and anti-BTLA Ab 20 μg/mouse, 80% of the paclitaxel and BP-1-102 40 μg/mouse group and 40% of the paclitaxel and LY294002 800 μg/mouse group were still alive 100 days after tumor challenge.

Furthermore, the effects of in vivo depletion of B cell were explored with anti-CD19 Ab. Mice treated with paclitaxel 6 mg/kg and anti-CD19 Ab 30 μg/mouse lived longer than those treated with paclitaxel or anti-CD19 Ab alone (*p* = 0.004, log-rank test, Additional file 5: Figure S5). All mice daily treated with paclitaxel and anti-BTLA Ab 20 μg/mouse and 60% of animals daily treated with paclitaxel and anti-CD19 Ab were still alive 100 days after tumor challenge.

Therefore, chemotherapy combined with BTLA-related inhibitors or antibody-mediated B cell depletion could generate different but more potent anti-tumor effects than chemotherapy alone.

#### EOC patients with detectable BTLA expression in cancerous tissues had poor prognosis

**Fig. 4:**
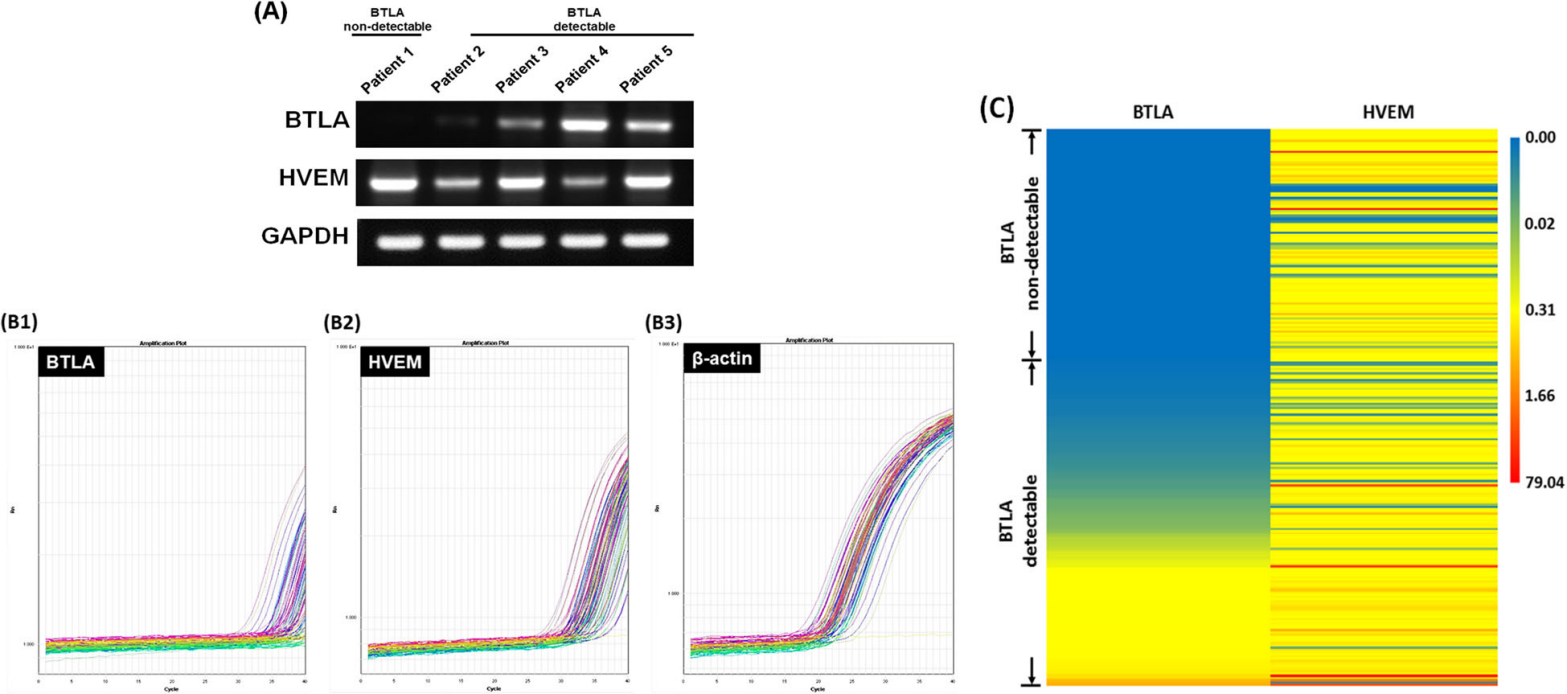
Expression of BTLA and HVEM in cancerous tissues of patients with EOCs. **a** Representative figures of the expressions of BTLA, HVEM, and GAPDH in cancerous tissues detected by RT-PCR. The expression of BTLA was not detected in all tissues of EOCs. **b** Representative figures of the expressions of BTLA **b1**, HVEM **b2** and β-actin **b3** in cancerous tissues analyzed by qPCR. The numbers of cycles for amplification-generated fluorescence to detect HVEM and β-actin were all within 40, but those to identify BTLA were not. **c** Heat map of complete results of BTLA and HVEM levels in 254 cancerous samples by qPCR. Each row represents a sample and each column represents BTLA or HVEM levels. Relative expression levels of the two molecules were shown by colors and intensities. The blue indicates low, yellow for median and red for high

The potential adverse effects of BTLA expression were preclinically demonstrated in animal model. Then, we conducted a survey to analyze BTLA and HVEM expression levels in cancerous samples of 254 EOC patients. As shown in Fig. 4a, unlike HVEM and GAPDH, BTLA expression could not be observed in all tissues of EOCs by RT-PCR. Similar findings were also obtained in the qPCR analysis, which represented the expression levels of BTLA, HVEM, and β-actin in the tissues (Fig. 4b). The numbers of cycles for amplification- generated fluorescence to identify HVEM and β-actin were all within 40, but the numbers for recognizing BTLA were not. Therefore, the 40 cycles of qPCR for detecting the BTLA were considered as a cut-off value to divide the studied population (254 women) into two sets, non-detectable BTLA (number of cycles ≥40, 105 cases) and detectable BTLA (number of cycles < 40, 149 cases) groups. Complete results of BTLA and HVEM expression levels in cancerous samples of 254 patients by qPCR were presented in Fig. 4c. Relative expression levels of these two molecules were shown by colors and intensities.

Based on the clinico-pathological characteristics of the 254 cases (Table 1), the mean follow-up duration was 38.2 months, and mean age at the time of disease diagnosis was 53.3 years. Distribution of disease status did not differ significantly between groups for histology, tumor grade, postoperative residual tumor size, or expression level of HVEM in cancerous tissues. However, patients in the detectable BTLA group had higher incidences of advanced disease (*p* = 0.025), disease relapse (*p* < 0.001), and disease-related death (*p* < 0.001) than those in the non-detectable BTLA group.

**Table 1:** Clinico-pathologic characteristics of the 254 EOC patients

Characteristic	BTLA		
	Non-detectable (*n* = 105)	Detectable (*n* = 149)	*p* value
Follow-up period [mean ± SD, months]	38.2 ± 31.5		
Age [mean ± SD, years]	53.3 ± 11.8		
FIGO stage^a^ [early/advanced, cases]	33/72	28/121	0.025
Histology^a^ [serous/mucinous/clear cell /endometrioid/undifferentiated, cases]	57/0/13/24/11	90/1/14/24/20	0.471
Tumor grade^a,c^ [1&2/3, cases]	16/86	19/124	0.712
Postoperative residual tumor^a^ [< 1 cm/≥1 cm, cases]	65/40	79/70	0.198
HVEM^b^ [median, range]	1.05 (0.01–79.0)	1.52 (0.04–78.2)	0.135
BTLA^b^ [median, range]	0	0.16 (0.007–24.3)	< 0.001
Disease recurrence^a^ [yes/no, cases]	48/57	108/41	< 0.001
Disease-related death^a^ [yes/no, cases]	18/87	59/90	< 0.001

Abbreviations: *EOC* epithelial ovarian carcinoma; *SD* standard deviation; *FIGO* International Federation of Gynecology and Obstetrics; *HVEM* herpesvirus entry mediator; *BTLA* B and T lymphocyte attenuator

^a^ By Chi-square test

^b^ By Mann-Whitney test

^c^ Data unavailable for nine patients

The prognostic factors for DFS of the studied population are shown in Table 2. By univariate analysis, advanced ovarian cancer [advanced versus early, HR: 3.6 (95% CI 2.2–5.8), *p* < 0.001], serous ovarian carcinoma [serous versus non-serous, HR: 1.5 (95% CI 1.1–2.2), *p* = 0.01], high-grade tumor [grade 3 versus grades 1–2, HR: 2.0 (95% CI 1.1–3.4), *p* = 0.015], ≥1 cm postoperative residual tumor [≥1 cm versus < 1 cm, HR: 2.8 (95% CI 2.1–3.9), *p* < 0.001], and detectable BTLA expression in cancerous tissue [detectable versus non-detectable, HR: 2.0 (95% CI 1.4–2.9), *p* < 0.001] were associated with significantly negative impacts on DFS. Advanced ovarian cancer [advanced versus early, HR: 2.3 (95% CI 1.3–4.2), *p* = 0.004], ≥1 cm postoperative residual tumor [≥1 cm versus < 1 cm, HR: 2.1 (95% CI 1.4–2.9), *p* < 0.001], and detectable BTLA expression in cancerous tissue [detectable versus non-detectable, HR: 1.7 (95% CI 1.2–2.4), *p* = 0.002] were independent prognostic factors for poor DFS in the 254 patients by multivariate analysis.

**Table 2:** Cox proportional hazards model for disease-free and overall survivals of 254 patients with EOC

DFS	Univariate HR (95% CI)^b^	*p*	Multivariate HR (95% CI)^b^	*p*	OS	Univariate HR (95% CI)^b^	*p*	Multivariate HR (95% CI)^b^	*p*
FIGO stage [advanced/early]	3.6 (2.2–5.8)	< 0.001	2.3 (1.3–4.2)	0.004	FIGO stage [advanced/early]	2.6 (1.3–4.8)	0.004	1.3 (0.6–2.7)	0.53
Histology [serous/non-serous^a^]	1.5 (1.1–2.2)	0.01	1.1 (0.8–1.6)	0.63	Histology [serous/non-serous^a^]	1.1 (0.7–1.7)	0.74		
Tumor grade [3/1–2]	2.0 (1.1–3.4)	0.015	1.6 (0.9–2.8)	0.11	Tumor grade [3/1–2]	1.4 (0.7–2.9)	0.39		
Postoperative residual tumor [≥1 cm/< 1 cm]	2.8 (2.1–3.9)	< 0.001	2.1 (1.4–2.9)	< 0.001	Postoperative residual tumor [> 1 cm/< 1 cm]	3.1 (2.0–5.0)	< 0.001	2.7 (1.6–4.7)	< 0.001
BTLA expression [detectable/non- detectable]	2.0 (1.4–2.9)	< 0.001	1.7 (1.2–2.4)	0.002	BTLA expression [detectable/non- detectable]	2.0 (1.2–3.5)	0.009	1.8 (1.04–3.0)	0.035

Abbreviations: *EOC* epithelial ovarian carcinoma; *DFS* disease-free survival; *OS* overall survival; *HR* Hazard ratio; *CI* confidence interval; *FIGO* International Federation of Gynecology and Obstetrics; *BTLA* B and T lymphocyte attenuator

^a^Non-serous includes mucinous, clear cell, endometrioid, and undifferentiated types

^b^Cox regression model

The prognostic factors for OS of the studied population were also analyzed (Table 2). By univariate analysis, advanced ovarian cancer [advanced versus early, HR: 2.6 (95% CI 1.3–4.8), *p* = 0.004], ≥1 cm postoperative residual tumor [≥1 cm versus < 1 cm, HR: 3.1 (95% CI 2.0–5.0), *p* < 0.001], and detectable BTLA expression in cancerous tissue [detectable versus non-detectable, HR: 2.5 (95% CI 1.2–3.5), *p* = 0.009] were significantly associated with negative impacts on OS. By multivariate analysis, ≥1 cm postoperative residual tumor [≥1 cm versus < 1 cm, HR: 2.7 (95% CI 1.6–4.7), *p* < 0.001] and detectable BTLA expression in cancerous tissue [detectable versus non-detectable, HR: 1.8 (95% CI 1.04–3.0), *p* = 0.035] were independent prognostic factors for poor OS.

Therefore, the adverse effects of BTLA expression on DFS or OS of patients with EOCs were clinically demonstrated from the analysis of cancerous tissues.

## Discussion

In this study, we evaluated the potential of BTLA to predict outcomes for EOC patients clinically and as targets for cancer treatment preclinically. Detectable BTLA expression in ovarian cancerous tissues was prognostic for poor outcomes for DFS and OS. The combination of chemotherapy and anti-BTLA Ab for inhibiting BTLA significantly reduced peritoneal tumor volumes and extended survival of tumor-bearing mice. In addition, BTLA could be mostly identified on B lymphocytes, especially on CD19^hi^ B cells, rather than on T lymphocytes and NK cells. Under regulation of IL-6 and IL-10, more BTLA^+^CD19^hi^ B lymphocytes could be induced through the AKT and STAT3 signaling pathways.

Many hallmarks of cancer are related to the TME, which is both a cause and consequence of tumorigenesis with the characteristics of tumor growth, invasion, and metastasis [34]. During tumorigenesis, various immune components, including immune checkpoints, are induced to create an immunosuppressive TME for escaping immune surveillance [5, 35]. In several studies, the expression levels of immune checkpoints CTLA4 or PD-L1 in tumors are reported to have prognostic utility for cancer patients [36–38]. High PD-L1 expression has been described to be a negative prognostic factor in ovarian cancer, and the PD-1/PD-L pathway can be a target for restoring antitumor immunity [39]. In this study, we investigated the prognostic utility of another immune checkpoint, BTLA, in EOC. The expression of BTLA was not detected in all 254 of the EOC specimens (Fig. 4). Cases with detectable BTLA expression had shorter DFS and OS than non-detectable cases (Table 2). However, in our studied population, the expression levels of BTLA had weak correlations with those of the other checkpoint molecules, including CTLA-4, PD-1, and PD-L1 (Additional file 6: Figure S6).

Currently, chemotherapy is a mainstream modality for treating EOCs. In addition to direct cancer killing activities, chemotherapy is reported to regulate anti-tumor T cell responses through increasing tumor antigenicity, inducing immunogenic cell death, disrupting immune suppressive pathways, and enhancing the effector T-cell response [40–43]. Initial response rates of patients with high-grade serous ovarian cancer, the most common subtype of EOCs, are 60–80%, but eventually most cases become chemoresistant with subsequent recurrences. One of the mechanisms for chemoresistance is related to the expression of the immune checkpoint in TME [44].

Recently, immune checkpoint inhibitors have provided promising clinical benefits in cancer treatment by elevating the anti-tumor immune responses of the patient’s immune system. These blockades have transformed therapeutics in various types of cancers, including melanoma, renal cell carcinoma, colorectal cancer, and non-small cell lung cancer [5, 45]. Several clinical trials of immune checkpoint blockades for treating EOC patients are also ongoing [44]. The mice treated with anti-BLTA Ab alone showed significant anti-tumor activities, which is resulted from inhibition of BTLA-mediated immunosuppression. And the anti-tumor effects were the same as those of paclitaxel-treated group (Fig. 1b and c). In addition, the immunologic changes of paclitaxel-treated group were similar to those of anti-BLTA Ab-treated group (Fig. 2a-d and f). The continuous low-dose (metronomic) administration of chemotherapeutic drugs has been reported to modulate the host immunity of tumor-bearing mice [46].

Consequently, the combination of immune checkpoint inhibitor with chemotherapy may increase the chemotherapeutic response, overcome immune suppression, and further generate potent anti-tumor effects with improved clinical outcomes. For this reason, we preclinically tested the chemotherapeutic agent paclitaxel in combination with an immune checkpoint inhibitor, anti-BTLA Ab, in an animal model of ovarian cancer (Fig. 1a). The combination of chemotherapy and anti-BTLA Ab significantly reduced peritoneal tumor volumes (Fig. 1b and c) and extended survival of tumor-bearing mice (Fig. 1d). The mice treated with chemotherapy and anti-BTLA Ab had higher percentages of activated CD4^+^ and C8^+^ T lymphocytes (Fig. 2a-d). Splenocytes from the tumor-bearing mice undergoing chemotherapy, incubated with anti-BTLA Ab, demonstrated greater cytotoxic effects (Fig. 2e). The concentrations of pro-inflammatory cytokines such as IL-12, TNF-α, and IFN-γ were higher in the ascites of tumor-bearing mice receiving chemotherapy combined with anti-BTLA Ab (Fig. 2f1-f3).

Ligand (antigen)-receptor (T and B cell receptor) interactions can generate the acquired anti-tumor immune response. Many of these ligands can interact with multiple receptors, some of which deliver co-activatory signals and others deliver inhibitory signals. In addition, another essential mechanism for activation of host immunities comes from the interactions of various cytokines in TME [5, 47]. However, immunosuppressive cytokines such as IL-10 and TGF-β can still induce PD-1 expression to limit immune surveillance [48, 49]. BTLA also has been reported as a suppressive pathway for T cell, B cell, or NKT cell-mediated immune responses [9, 12–15]. This molecule can be expressed on T cells, B cells, NK cells, and the other cells [50]. Based on our study, BTLA expression was more abundant on B lymphocytes than that on T lymphocytes or NK cells. When these B lymphocytes were further subclassified, BTLA was largely identified on the CD19^hi^ B lymphocytes (Fig. 3a). Nevertheless, few studies are available to evaluate the regulation of BTLA expression.

Alterations in percentages of BTLA^+^CD19^hi^ B lymphocytes in splenocytes (Fig. 3b) and TACs of ascites (Fig. 3c) revealed the role of BTLA expression in tumor progression. The disparity was more obvious in TACs of ascites, which was part of TME [25, 26]. Based on our previous report [24], elevated anti-inflammatory cytokines such as IL-6, IL-10, and TGF-β in tumor-associated ascites are related to tumor progression. Consequently, we further investigated the relationship between anti-inflammatory cytokines and BTLA expression. As shown here, BTLA^+^CD19^hi^ B lymphocytes can be induced under regulation of IL-6 and IL-10 (Fig. 3d). AKT and STAT3 signaling pathways were involved in the control of BTLA expression (Fig. 3e). In addition to anti-BTLA Ab, chemotherapy combined with other BTLA-related inhibitors such as LY294002 (AKT inhibitor) or BP-1-102 (STAT3 inhibitor) can generate potent anti-tumor effects compared to chemotherapy alone (Fig. 3f). However, the anti-tumor effects of paclitaxel combined with various BTLA-related inhibitors were different. Our explanation might be their specificity. Anti-BTLA Ab can be more specific to target BTLA exclusively and then inhibit the functions of BTLA. Besides, the other two molecules, AKT and STAT3, are not only involved in regulating BTLA expression but also modulating several signaling pathways in the process of tumor progression.

## Conclusions

In conclusion, BTLA can be predictive of poor outcome in EOC. In addition, IL-6 and IL-10 can induce the percentages of BTLA^+^CD19^hi^ B lymphocytes through AKT and STAT3 signaling pathways in TMEs. Furthermore, inhibition of BTLA combined with chemotherapy can promote immune activation and generate potent anti-tumor effects in an animal model. Therefore, the combination of chemotherapy and anti-BTLA Ab for treating cancer may hold clinical potential.

## Supplementary information

**Additional file 1: Figure S1.** Tumor re-challenge of the surviving mice. All mice daily treated with paclitaxel 6 mg/kg and anti-BTLA Ab 20 μg/mouse were alive 100 days after tumor challenge. The therapy was discontinued on day 100 and the mice were subcutaneously re-challenged with 1 × 10^5^ WF-3/Luc tumor cells. Subcutaneous (yellow cycle) tumors of mice can be detected by IVIS system (5 mice in this analysis).

**Additional file 2: Figure S2.** Survival analysis of tumor-bearing mice undergoing various therapeutic agents combined with anti-BTLA Ab. (A) Survival analysis of mice treated with cisplatin and anti-BTLA Ab. Mice daily treated with cisplatin 1 mg/kg and anti-BTLA Ab 20 μg/mouse had longer survival intervals than those daily treated with cisplatin 1 mg/kg or anti-BTLA Ab 20 μg/mouse alone (*p* = 0.02, log-rank test). (5 mice in each group) **(B)** Survival analysis of mice treated with bevacizumab and anti-BTLA Ab. Mice daily treated with bevacizumab 2 mg/kg and anti-BTLA Ab 20 μg/mouse had longer survival intervals than those daily treated with bevacizumab 2 mg/kg or anti-BTLA Ab 20 μg/mouse alone (*p* < 0.001, log-rank test). Sixty percent of animals treated with bevacizumab and anti-BTLA Ab were alive 100 days after tumor challenge. (5 mice in each group) **(C)** Survival analysis of mice treated with olaparib and anti-BTLA Ab. Mice daily treated with olaparib 5 mg/kg and anti-BTLA Ab 20 μg/mouse had longer survival intervals than those daily treated with olaparib 5 mg/kg or anti-BTLA Ab 20 μg/mouse alone (*p* = 0.01, log-rank test). Forty percent of mice treated with olaparib and anti-BTLA Ab were alive 100 days after tumor challenge. (5 mice in each group).

**Additional file 3: Figure S3.** Survival analysis of tumor-bearing mice received chemotherapy combined with various immune checkpoint blockades. (A) Survival analysis of mice treated with respective immune checkpoint blockade alone. Survivals of mice daily treated with paclitaxel 6 mg/kg, anti-BTLA Ab 20 μg/mouse, anti-PD-1 Ab 30 μg/mouse, or anti-PD-L1 Ab 30 μg/mouse alone did not show difference (*p* = 0.39, log-rank test). (5 mice in each group) **(B)** Survival analysis of mice treated with chemotherapy and various immune checkpoint blockades. Sixty percent of mice daily treated with paclitaxel and anti-PD-L1 Ab and 80% of mice daily treated with paclitaxel and anti-PD-1 Ab were alive 100 days after tumor challenge. All mice daily treated with paclitaxel and anti-BTLA Ab, paclitaxel, anti-PD-1 Ab and anti-BTLA Ab, or paclitaxel, anti-PD-L1 Ab and anti-BTLA Ab were alive 100 days after tumor challenge. (5 mice in each group).

**Additional file 4: Figure S4.** Kinetic alterations of BTLA^+^CD19^hi^ B lymphocytes in tumor microenvironment of tumor-bearing mice after different days of tumor challenge. (A) Representative flow cytometric figures of the percentages of BTLA^+^CD19^hi^ B lymphocytes in TILs on indicated days after tumor challenge. (5 mice in each group) **(B)** Bar figures of the percentages of BTLA^+^CD19^hi^ B lymphocytes in TILs on day 14 or day 35 after tumor challenge. The percentages of BTLA^+^CD19^hi^ B lymphocytes were higher on day 35 (18.18 ± 0.65%) than on day 14 (5.46 ± 0.58%) (*p* = 0.009, Kruskal-Wallis test). (5 mice in each group).

**Additional file 5: Figure S5.** Survival analysis of tumor-bearing mice treated with chemotherapy and B cell depletion with anti-CD19 Ab. Mice daily treated with paclitaxel 6 mg/kg and anti-CD19 Ab 30 μg/mouse lived longer than those daily treated with paclitaxel or anti-CD19 Ab alone (*p* = 0.004, log-rank test). All mice daily treated with paclitaxel and anti-BTLA Ab 20 μg/mouse and 60% of animals daily treated with paclitaxel and anti-CD19 Ab were alive 100 days after tumor challenge. (5 mice in each group).

**Additional file 6: Figure S6.** Correlations of various immune checkpoint molecules in ovarian cancerous tissues. The expression levels of BTLA had weak correlations (correlation *coefficient, R < 0.4*) with those of CTLA-4, PD-1, and PD-L1. Whereas, the expression levels of CTLA-4, PD-1, and PD-L1 had high correlations (correlation *coefficient, R ≥ 0.4*) in ovarian cancerous tissues.

## Data Availability

All of the patients’ personal data were fully anonymized before analysis.
