# Supplementary figures and images for: BTLA blockade enhances Cancer therapy by inhibiting IL-6/IL-10-induced CD19high B lymphocytes
Source: J Immunother Cancer. 2019 Nov 21;7:313. doi: 10.1186/s40425-019-0744-4 (PMC6868712; doi:10.1186/s40425-019-0744-4)

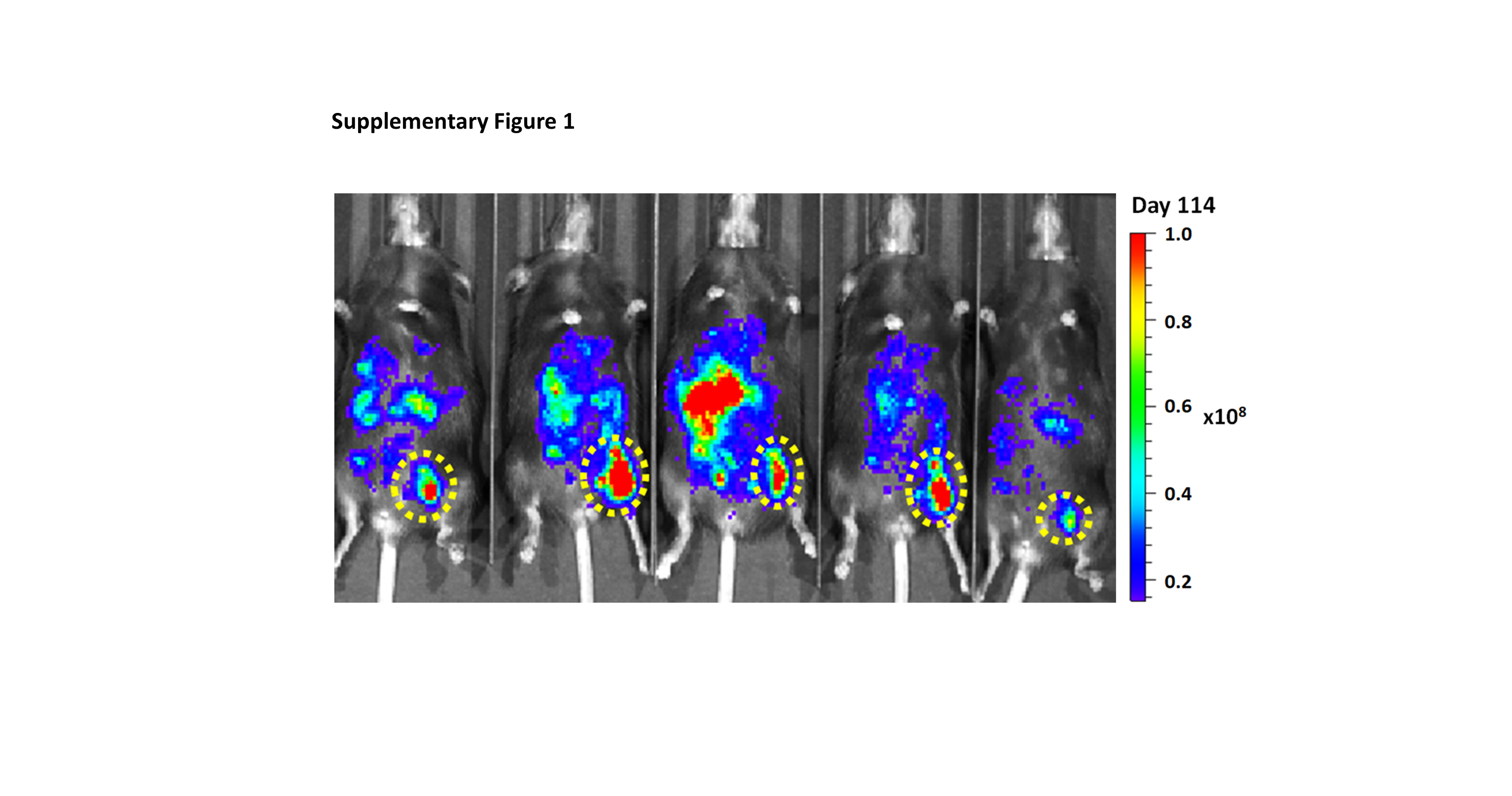

Supplement: Supplementary file 1 — Additional file 1: Figure S1. Tumor re-challenge of the surviving mice. All mice daily treated with paclitaxel 6 mg/kg and anti-BTLA Ab 20 μg/mouse were alive 100 days after tumor challenge. The therapy was discontinued on day 100 and the mice were subcutaneously re-challenged with 1 × 10^5^ WF-3/Luc tumor cells. Subcutaneous (yellow cycle) tumors of mice can be detected by IVIS system (5 mice in this analysis). [file 40425_2019_744_MOESM1_ESM.tif]

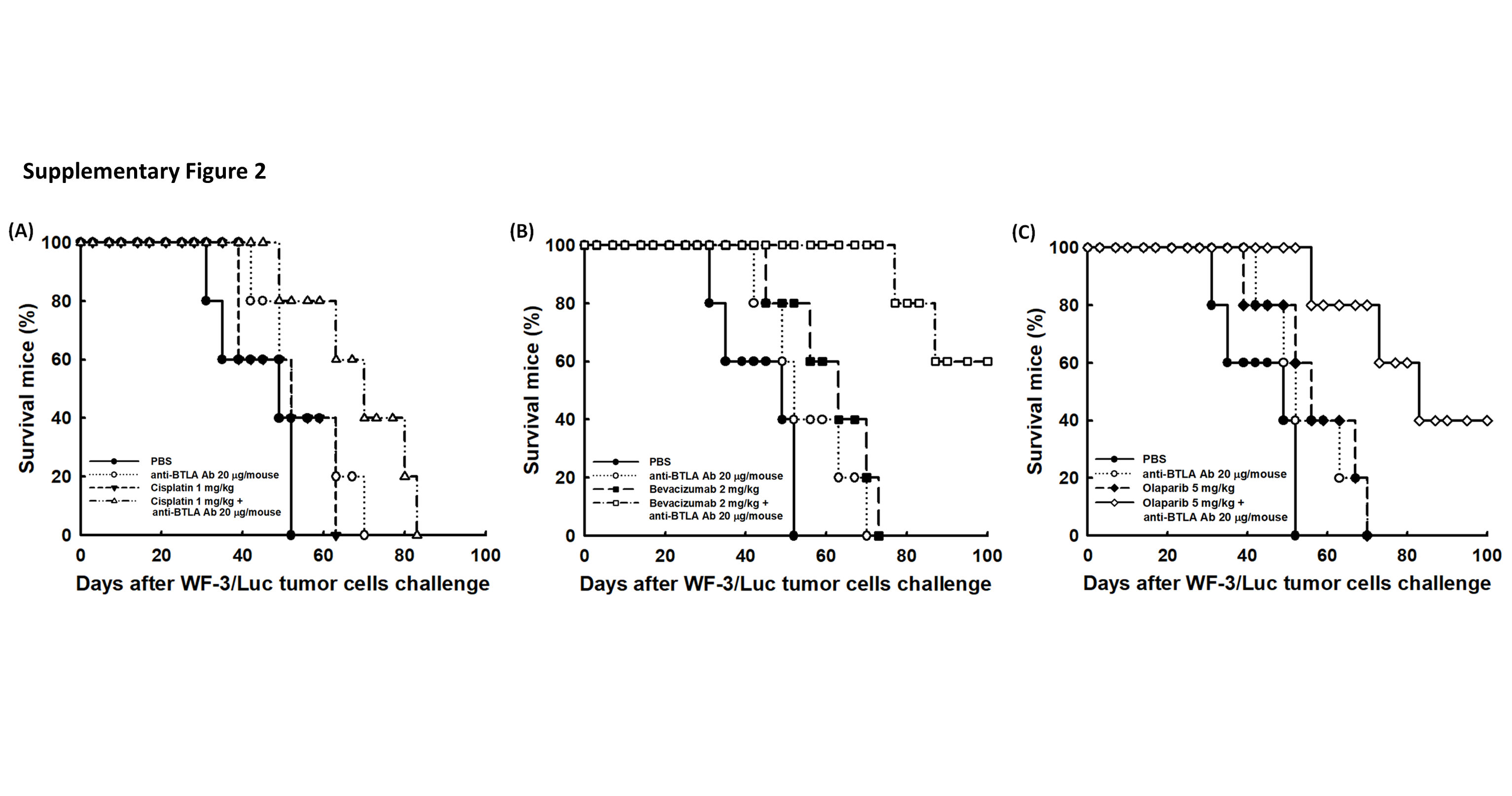

Supplement: Supplementary file 2 — Additional file 2: Figure S2. Survival analysis of tumor-bearing mice undergoing various therapeutic agents combined with anti-BTLA Ab. (A) Survival analysis of mice treated with cisplatin and anti-BTLA Ab. Mice daily treated with cisplatin 1 mg/kg and anti-BTLA Ab 20 μg/mouse had longer survival intervals than those daily treated with cisplatin 1 mg/kg or anti-BTLA Ab 20 μg/mouse alone (p = 0.02, log-rank test). (5 mice in each group) (B) Survival analysis of mice treated with bevacizumab and anti-BTLA Ab. Mice daily treated with bevacizumab 2 mg/kg and anti-BTLA Ab 20 μg/mouse had longer survival intervals than those daily treated with bevacizumab 2 mg/kg or anti-BTLA Ab 20 μg/mouse alone (p < 0.001, log-rank test). Sixty percent of animals treated with bevacizumab and anti-BTLA Ab were alive 100 days after tumor challenge. (5 mice in each group) (C) Survival analysis of mice treated with olaparib and anti-BTLA Ab. Mice daily treated with olaparib 5 mg/kg and anti-BTLA Ab 20 μg/mouse had longer survival intervals than those daily treated with olaparib 5 mg/kg or anti-BTLA Ab 20 μg/mouse alone (p = 0.01, log-rank test). Forty percent of mice treated with olaparib and anti-BTLA Ab were alive 100 days after tumor challenge. (5 mice in each group). [file 40425_2019_744_MOESM2_ESM.tif]

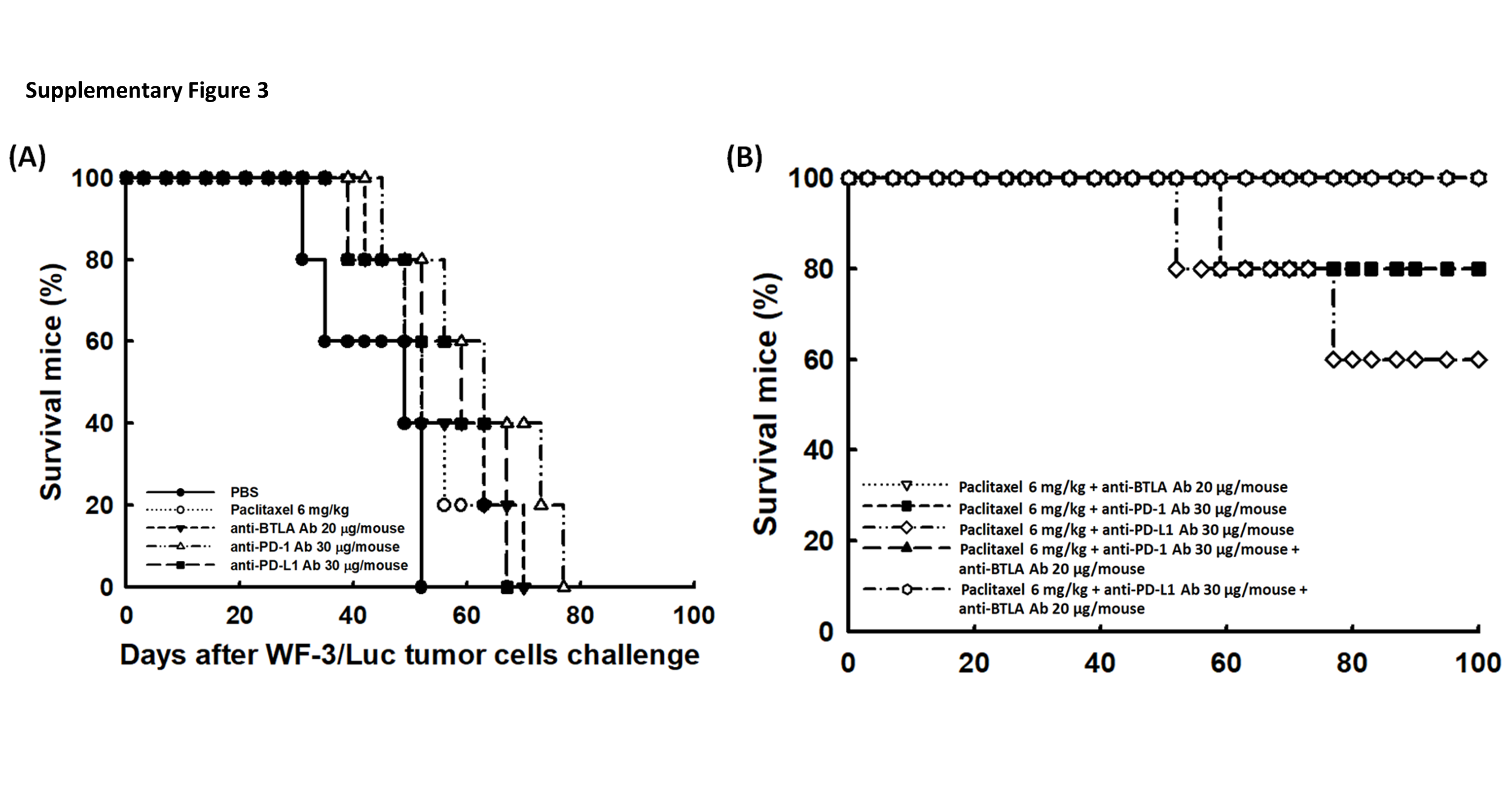

Supplement: Supplementary file 3 — Additional file 3: Figure S3. Survival analysis of tumor-bearing mice received chemotherapy combined with various immune checkpoint blockades. (A) Survival analysis of mice treated with respective immune checkpoint blockade alone. Survivals of mice daily treated with paclitaxel 6 mg/kg, anti-BTLA Ab 20 μg/mouse, anti-PD-1 Ab 30 μg/mouse, or anti-PD-L1 Ab 30 μg/mouse alone did not show difference (p = 0.39, log-rank test). (5 mice in each group) (B) Survival analysis of mice treated with chemotherapy and various immune checkpoint blockades. Sixty percent of mice daily treated with paclitaxel and anti-PD-L1 Ab and 80% of mice daily treated with paclitaxel and anti-PD-1 Ab were alive 100 days after tumor challenge. All mice daily treated with paclitaxel and anti-BTLA Ab, paclitaxel, anti-PD-1 Ab and anti-BTLA Ab, or paclitaxel, anti-PD-L1 Ab and anti-BTLA Ab were alive 100 days after tumor challenge. (5 mice in each group). [file 40425_2019_744_MOESM3_ESM.tif]

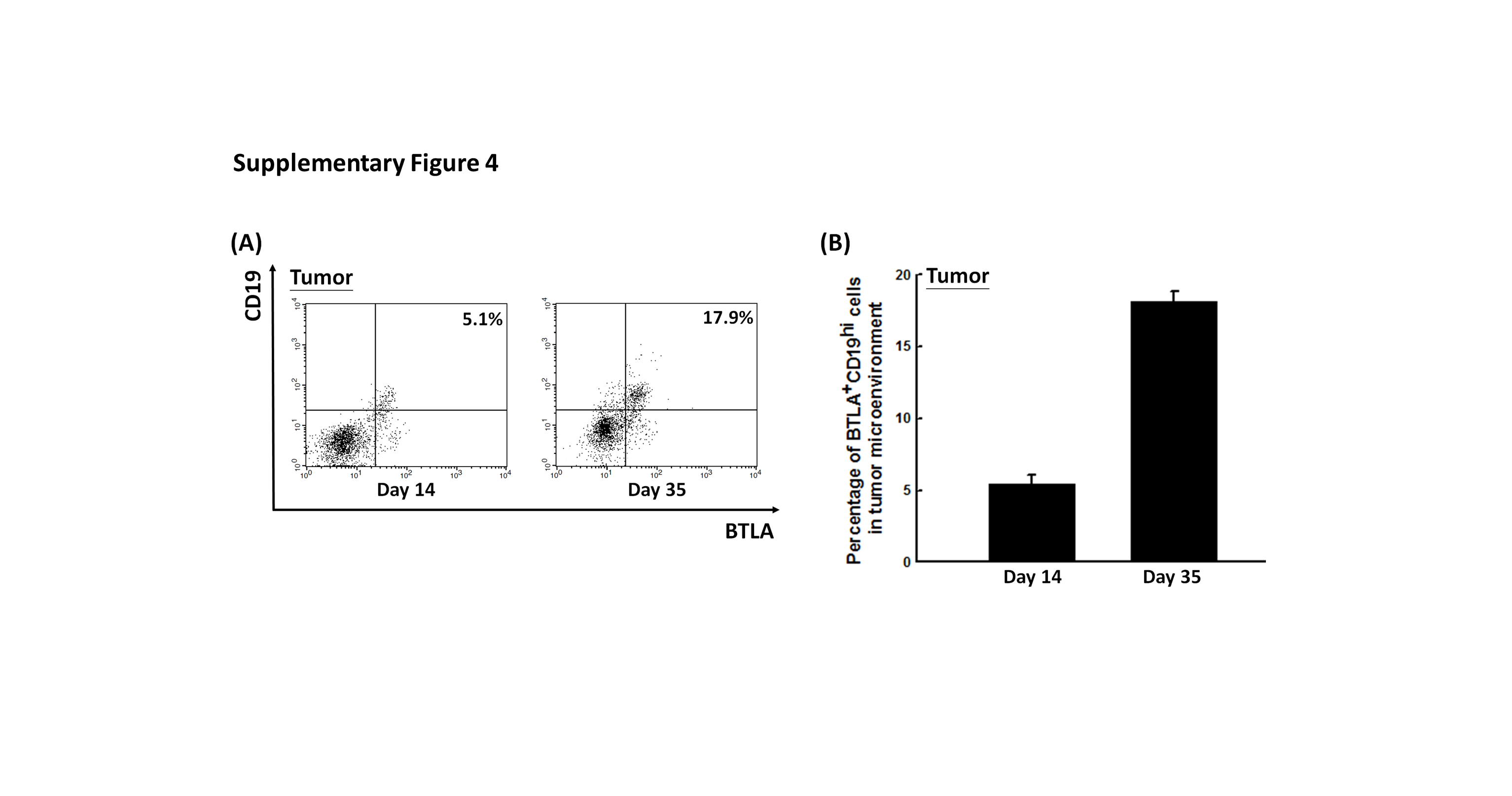

Supplement: Supplementary file 4 — Additional file 4: Figure S4. Kinetic alterations of BTLA+CD19hi B lymphocytes in tumor microenvironment of tumor-bearing mice after different days of tumor challenge. (A) Representative flow cytometric figures of the percentages of BTLA+CD19hi B lymphocytes in TILs on indicated days after tumor challenge. (5 mice in each group) (B) Bar figures of the percentages of BTLA+CD19hi B lymphocytes in TILs on day 14 or day 35 after tumor challenge. The percentages of BTLA+CD19hi B lymphocytes were higher on day 35 (18.18 ± 0.65%) than on day 14 (5.46 ± 0.58%) (p = 0.009, Kruskal-Wallis test). (5 mice in each group). [file 40425_2019_744_MOESM4_ESM.tif]

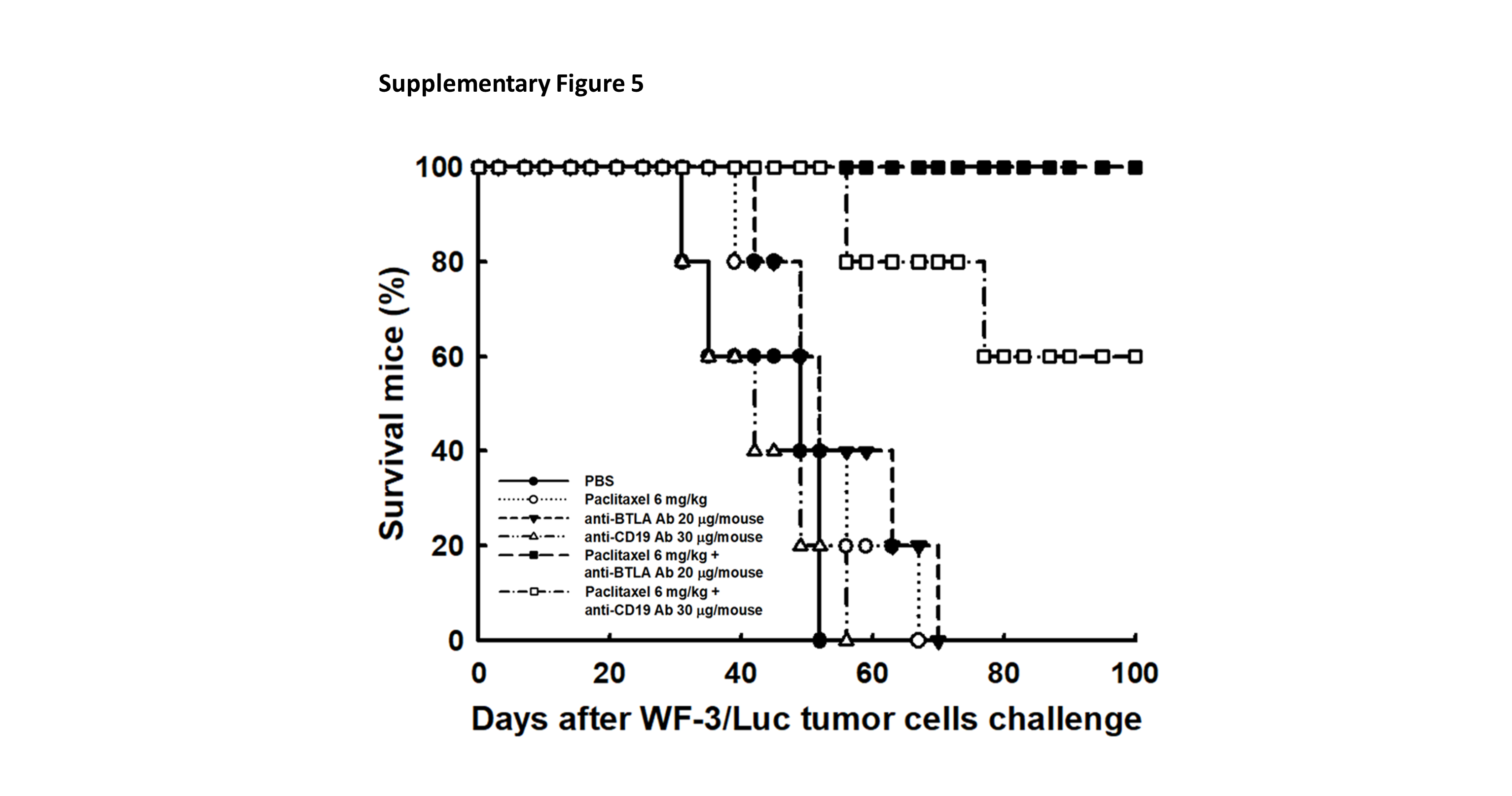

Supplement: Supplementary file 5 — Additional file 5: Figure S5. Survival analysis of tumor-bearing mice treated with chemotherapy and B cell depletion with anti-CD19 Ab. Mice daily treated with paclitaxel 6 mg/kg and anti-CD19 Ab 30 μg/mouse lived longer than those daily treated with paclitaxel or anti-CD19 Ab alone (p = 0.004, log-rank test). All mice daily treated with paclitaxel and anti-BTLA Ab 20 μg/mouse and 60% of animals daily treated with paclitaxel and anti-CD19 Ab were alive 100 days after tumor challenge. (5 mice in each group). [file 40425_2019_744_MOESM5_ESM.tif]

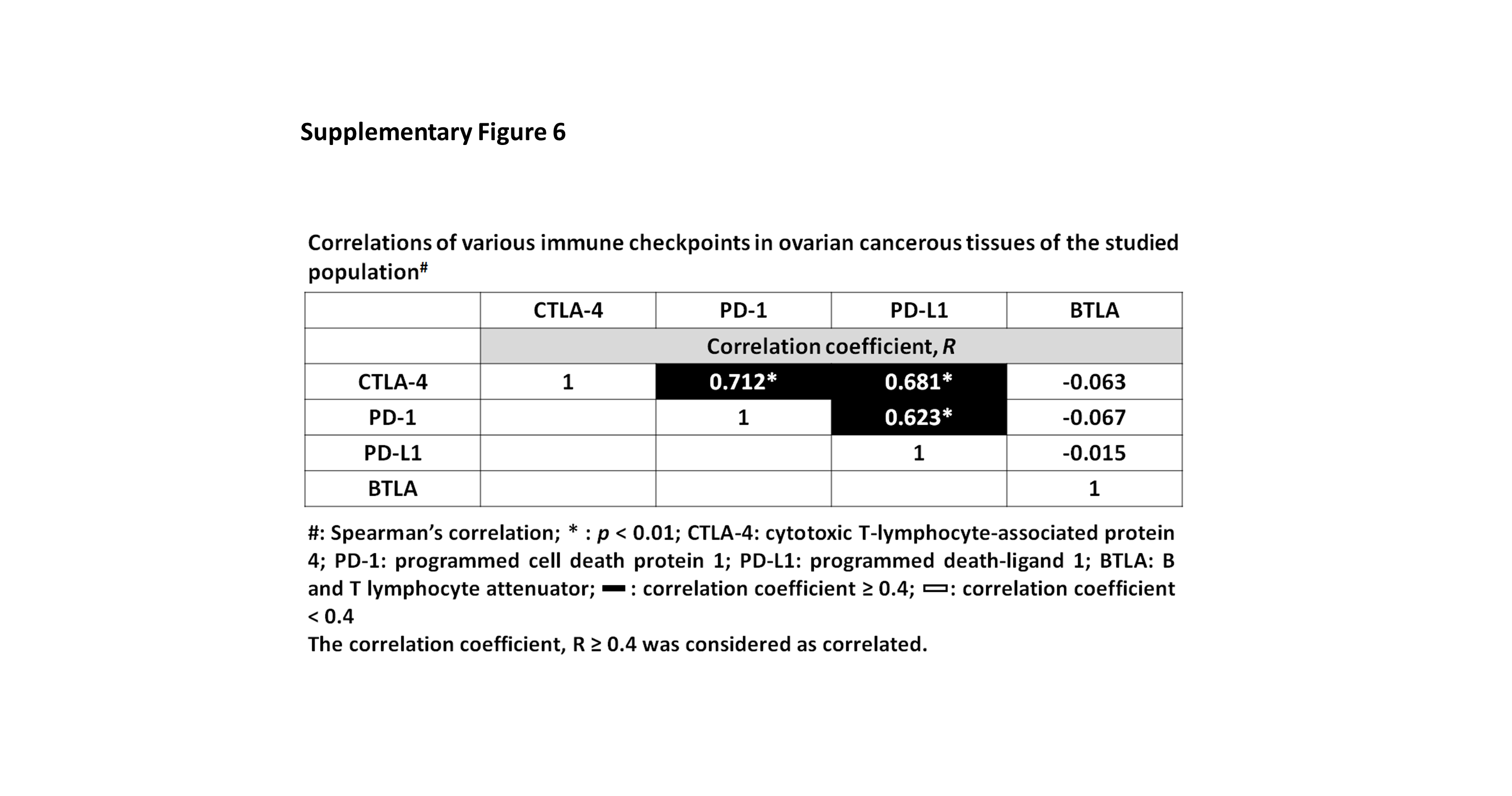

Supplement: Supplementary file 6 — Additional file 6: Figure S6. Correlations of various immune checkpoint molecules in ovarian cancerous tissues. The expression levels of BTLA had weak correlations (correlation coefficient, R < 0.4) with those of CTLA-4, PD-1, and PD-L1. Whereas, the expression levels of CTLA-4, PD-1, and PD-L1 had high correlations (correlation coefficient, R ≥ 0.4) in ovarian cancerous tissues. [file 40425_2019_744_MOESM6_ESM.tif]
